# Relationship between scapular control during isometric shoulder flexion and scapular motion during baseball pitching: a cross-sectional study

**DOI:** 10.1186/s13102-022-00471-9

**Published:** 2022-04-28

**Authors:** Yuki Nomura, Hajime Toda, Masaki Katayose, Shun Watanabe, Masahiro Yoshida, Makoto Yoshida, Keizo Yamamoto

**Affiliations:** 1 Graduate School of Health Sciences, Sapporo Medical University, West 11, South 5, Chuo-ku, Sapporo City, 060-8556 Japan; 2 Department of Rehabilitation, Sapporo Maruyama Orthopedic Hospital, 1-3, West 27, North 7, Chuo-ku, Sapporo City, 060-0007 Japan; 3 School of Health Sciences, Sapporo Medical University, West 11, South 5, Chuo-ku, Sapporo City, 060-8556 Japan; 4 School of Lifelong Sport, Hokusho University, 23, Bunkyodai, Ebetsu City, 069-8511 Japan

**Keywords:** Shoulder, Scapular motion, Throwing motion, Kinematics, Maximum shoulder external rotation

## Abstract

**Background:**

A baseball pitcher with decreased scapular control may not be able to achieve suitable scapular motion at maximum shoulder external rotation (MER) of baseball pitching during the pitching action. It is common clinically to compare scapular control of the throwing and non-throwing arms to detect side-to-side differences. However, it remains unclear whether scapular control is different between the throwing and non-throwing arms. Moreover, no data exist on the relationship between scapular control and scapular motion at MER of pitching. Primarily, this study aimed to compare scapular control during isometric shoulder flexion between the throwing and non-throwing arms. Secondly, this study aimed to investigate the relationship between scapular control during isometric shoulder flexion and scapular motion at MER of pitching.

**Methods:**

Fifteen healthy collegiate baseball pitchers (age, 20.2 ± 1.9 years; height, 1.76 ± 0.05 m; body mass, 73.3 ± 6.7 kg) were recruited. An optical motion tracking system was used to assess scapular motion. Scapular control was defined as the amount of change in the scapular internal rotation angle, downward rotation angle, and anterior tilt angle during isometric shoulder flexion. We assessed scapular position at MER of pitching.

**Results:**

No significant differences were detected for any of the scapular angles during isometric shoulder flexion between the throwing and non-throwing arms. The amount of change in the scapular internal rotation angle, scapular downward rotation angle, and scapular anterior tilt angle during isometric shoulder flexion had a significant relationship with the scapular downward rotation angle at MER.

**Conclusions:**

No side-to-side difference was noted in scapular control during isometric shoulder flexion in healthy collegiate baseball pitchers at the group level. Further studies are required to understand the side-to-side differences at the individual level. Additionally, there was a relationship between scapular control during isometric shoulder flexion and scapular position at MER. These findings suggest that clinicians may consider using isometric shoulder flexion to assess scapular control in baseball pitchers.

## Background

Abnormal scapular kinematics are a common cause of shoulder injuries in overhead athletes [1, 2]. Previous studies have reported that abnormal scapular kinematics during upper extremity movement occurs in patients with SLAP lesions [3], subacromial impingement syndrome [4, 5], and internal impingement of the shoulder [6, 7]. In particular, Mihata et al. [7] reported that an increased scapular internal rotation angle and downward rotation angle at the maximum shoulder external rotation (MER) of simulated baseball pitching caused internal impingement of the shoulder in vitro. It has been theoretically recognized that the ability to control the scapula against the thoracic cage is necessary for baseball pitchers to avoid abnormal scapular kinematics at MER during baseball pitching [8]. Thus, scapular control plays an important role in the normal biomechanics of pitching.

Several authors have emphasized the importance of appropriate co-contraction of the periscapular muscles in contributing to scapular control and developed specific exercises to enhance neuromuscular control of the scapula [9–13]. In these exercises, the patient is asked to stabilize the scapula to the thoracic cage against the clinician’s manual resistance during isometric contraction of the shoulder muscle. It is generally believed that scapular control exercise is necessary for baseball pitchers [13]. Additionally, previous studies have shown that individuals with decreased scapular control have excessive scapular internal rotation, downward rotation, and anterior tilt during isometric shoulder flexion [14–17]. Therefore, clinicians should consider evaluating the amount of change in the scapular position during isometric shoulder flexion as the ability to control the scapula. A pitcher with decreased scapular control during isometric shoulder flexion may not be able to achieve suitable scapular motion during baseball pitching.

It is common clinically to assess the resting scapular position and dynamic scapular motion of the throwing and non-throwing arms to detect side-to-side differences [8]. The amount of change in scapular position during isometric shoulder flexion is accentuated with a load [16]. However, whether scapular control during isometric shoulder flexion is different between the throwing and non-throwing arms remains unclear. Moreover, no data exist on the relationship between scapular control during isometric shoulder flexion and scapular motion during baseball pitching.

The primary aim of this study was to compare scapular control during isometric shoulder flexion between the throwing and non-throwing arms. Previous research has shown that shoulder flexion strength was not different between the throwing and non-throwing arms [18]. Therefore, we hypothesized that scapular control during isometric shoulder flexion would not be different between the throwing and non-throwing arms. The secondary aim of this study was to investigate the relationship between scapular control during isometric shoulder flexion and scapular position at MER during baseball pitching. We hypothesized that there would be a relationship between the increased scapular internal rotation angle, downward rotation angle, and anterior tilt angle during isometric shoulder flexion and the scapular internal rotation angle and downward rotation angle at MER during baseball pitching. Direct examination of the relationship between scapular control during isometric shoulder flexion and scapular motion during pitching may be crucial in improving scapular motion during baseball pitching.

## Methods

### Ethics statements

All participants provided written informed consent prior to participating in the experiments. The Sapporo Medical University Ethical Committee (30-2-2) and Hokusho University Ethical Committee (2017-014) approved this study, and the study’s ethical aspects conformed to the principles of the Declaration of Helsinki.

### Study design and participants

Fifteen male college baseball pitchers (age, 20.2 ± 1.9 years; height, 1.76 ± 0.05 m; body mass, 73.3 ± 6.7 kg; throwing arm, 4 left and 11 right) were recruited for this cross-sectional study. All pitchers were recruited from the same college baseball team and used an overhand style during throwing. Participants were required to have more than 3 years of experience in baseball pitching. Pitchers were excluded if they had pain in the upper/lower extremities and trunk at the time of testing or had a history of upper/lower extremity and trunk surgery at least 3 years before the day of data collection.

### Instrumentation

**Fig. 1:**
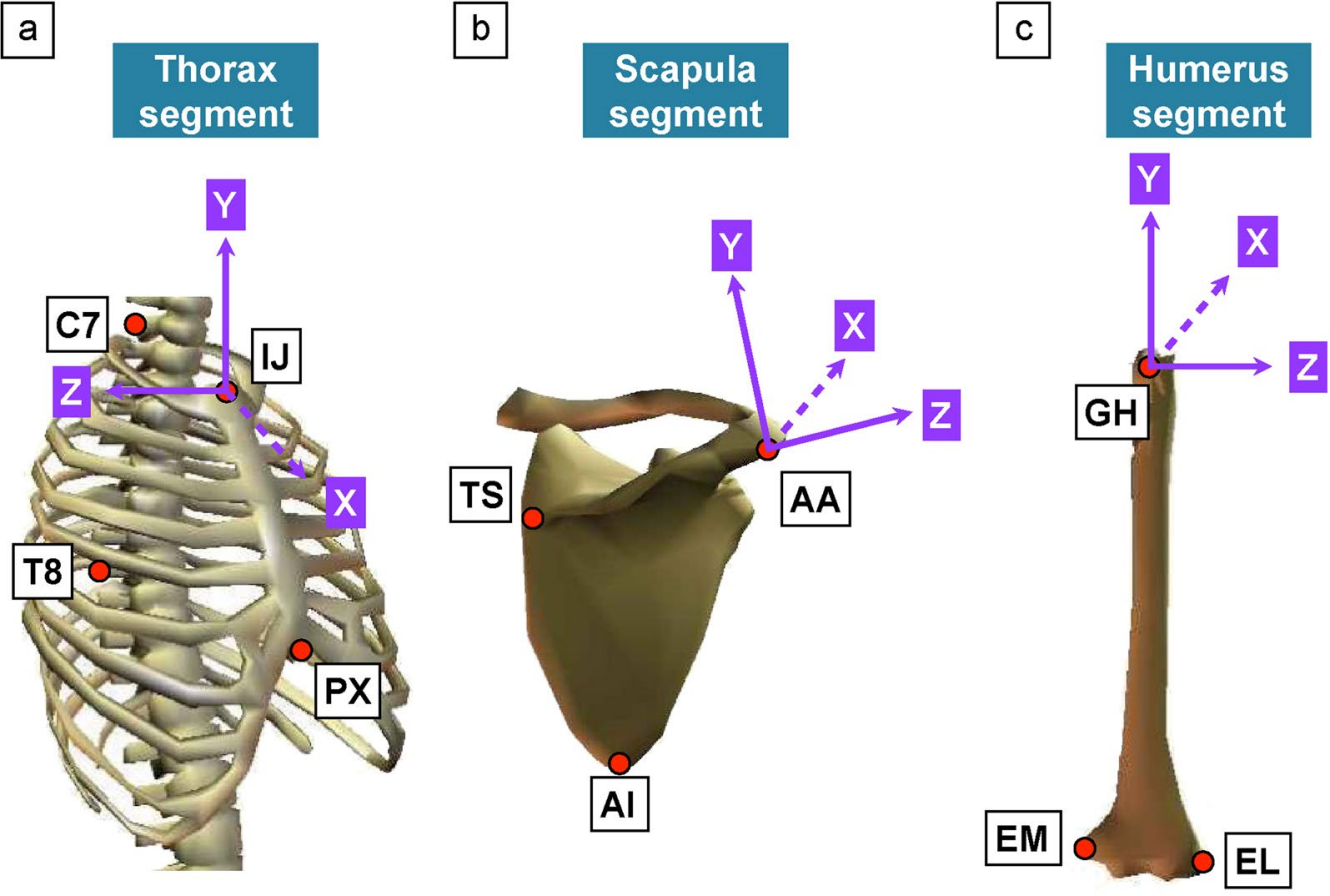
Anatomical bony landmarks and the defined local coordinate system. **a** To define the thorax coordinate systems, we attached four markers to the spinous process of the seventh cervical vertebra (C7), spinal process of the eighth thoracic vertebra (T8), incisura jugularis (IJ), and processus xiphoideus (PX). **b** To define the scapula coordinate systems, we attached three markers to the trigonum spinae scapulae (TS), angulus inferior (AI), and angulus acromialis (AA). **c** To define the humerus coordinate systems, we attached three markers to the most caudal point on the lateral epicondyle (EL), most caudal point on the medial epicondyle (EM), and the acromioclavicular (AC) joint. The glenohumeral joint center was estimated by calculating the marker position of the AC joint (GH)

**Fig. 2:**
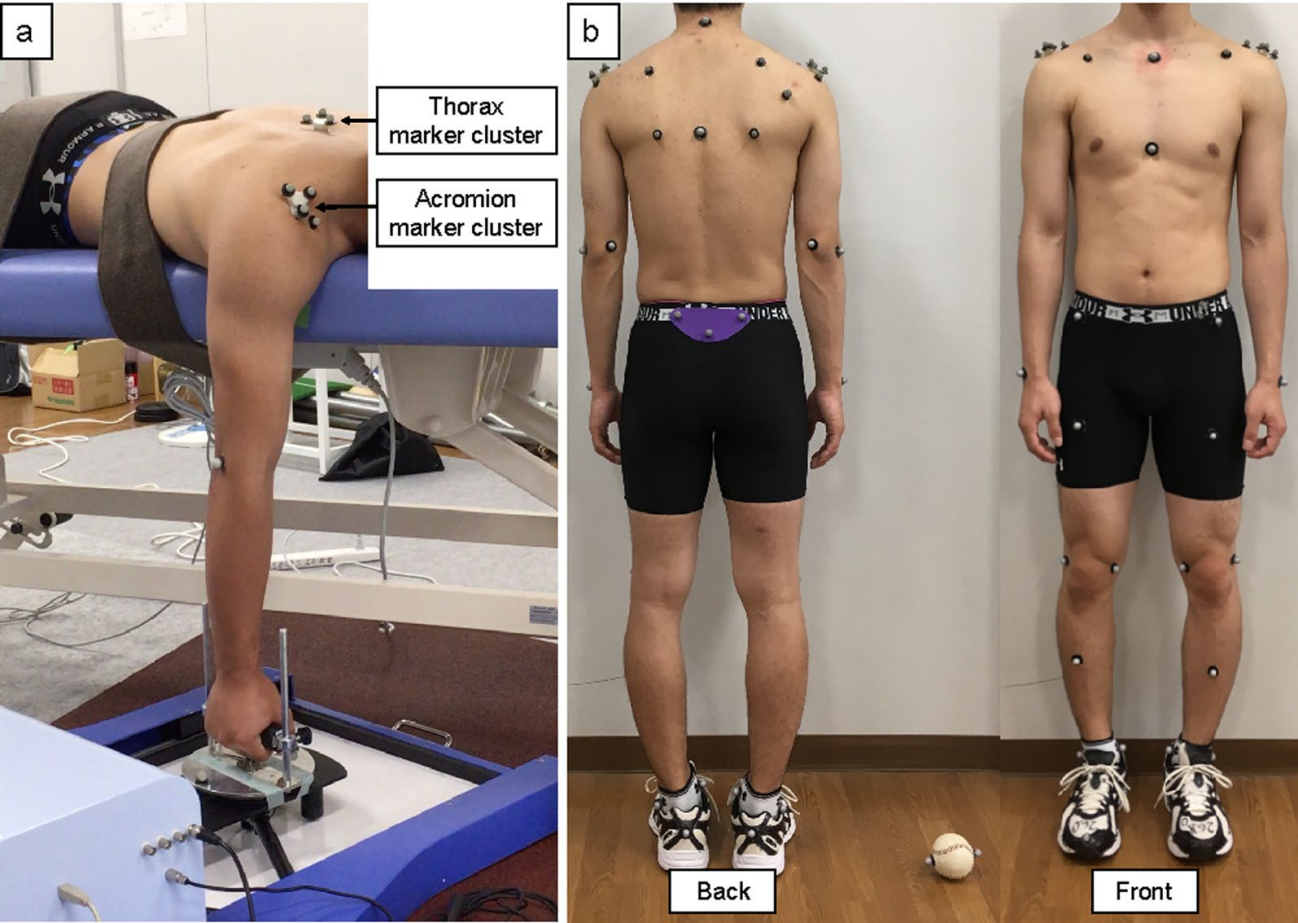
Location of the marker set. **a** The acromion and thorax marker cluster. **b** The marker set for baseball pitching analysis

Three-dimensional (3D) kinematic data of the scapular angle during isometric shoulder flexion were collected using a 12-camera motion capture system (MAC3D, Motion Analysis Corporation, Rohnert Park, CA) at 100 Hz. A set of 16 retro-reflective markers was used to track the thoracic and scapular segments [19] (Fig. 1a–c). One marker was attached to the acromioclavicular (AC) joint to define the glenohumeral joint center [20, 21], the acromion marker cluster to the flat part of the acromion [22–24], and the thorax marker cluster to the middle point between the spinous process of the seventh cervical vertebra and the spinal process of the eighth thoracic vertebra (Fig. 2a). The acromion and thorax marker cluster consisted of a base with three reflective markers. The static standing posture was recorded to link the position of the acromion marker cluster and the thorax marker cluster to local anatomical coordinate systems according to the ISB recommendations for the upper extremity [19]. From the combination of local anatomical coordinate systems and marker cluster motions, it was possible to calculate the scapular angles during dynamic shoulder motion [19, 24]. The acromion marker cluster method is a valid method of measuring scapular motion [22, 24]. This method has a measurement error of 3.5°–9.5°, and the advantages of non-invasiveness and less movement restriction. Kinematic data during baseball pitching were collected at 500 Hz, and a force plate (1000 Hz; BP6001200, AMTI, Watertown, MA) synchronized with the motion capture system was used to define the stride foot contact. A set of 16 retro-reflective markers [19] and acromion marker cluster [24] were used to track the thorax, scapula, and humerus segments. Moreover, we used the Helen Hayes marker set [25] to track the lower leg. Additionally, we attached two markers to the ball and one marker to the middle point between the styloid process of the ulna and radius in the dorsal wrist (wrist marker) to define the pitching phases (Fig. 2b). Rotational joint angles were calculated using the Euler angle method, which represents the difference in the orientation of each segment (Fig. 1). According to the ISB recommendations [19], we used the Y–X–Z′ sequence (internal rotation/external rotation, downward rotation/upward rotation, and posterior tilt/anterior tilt) for scapular rotations relative to the thorax and the Y–X–Y′ sequence (horizontal adduction/horizontal abduction, depression/elevation, internal rotation/external rotation) for shoulder rotations when looking at shoulder relative to thorax.

### Testing procedure

The isometric shoulder flexion torque (Nm) was measured for each participant before assessing scapular control during isometric shoulder flexion. This torque was calculated by multiplying the value of the maximum isometric muscle strength of shoulder flexion (N) by the value of the upper limb length (m). During the measurement of muscle strength, the participant was seated with the dominant (throwing) arm positioned at 90° of shoulder flexion with the thumb pointing in an upward direction, and instructed to perform a 3-s maximum voluntary isometric contraction after the ramp-up contraction. The hand-held dynamometer (Mobie, Sakai Medical, Tokyo, Japan) was positioned on the arm just proximal to the elbow, and the resistance was applied in a downward direction perpendicular to the humerus by the same examiner. The muscle strength was measured twice, and the highest value was used in the analysis. The time interval between the trials was 1 min. When all two trials of the throwing arm were completed, each participant performed testing of the non-throwing arm. The length of the upper limb was measured by the same examiner using a tape measure and was defined as the distance from the acromioclavicular joint to the styloid process of the radius.

**Fig. 3:**
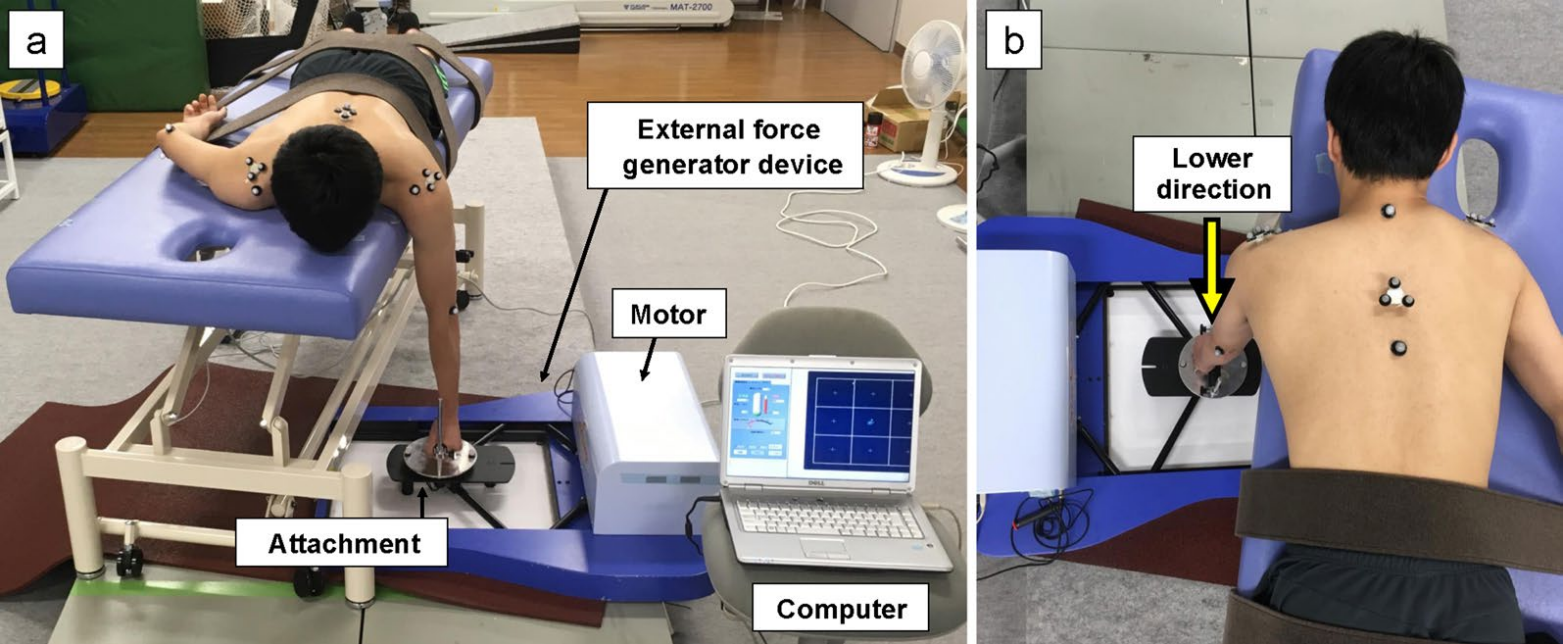
Assessing scapular control during isometric shoulder flexion. **a** The external force is applied to the participant by using an external force generator device. The attachment is connected to the motor of the external force generator device. The motor is controlled by a personal computer connected to the device. **b** The direction of the external force is defined as the lower direction (the external force is directed from the cranial to the caudal direction)

To assess scapular control during isometric shoulder flexion, an external force was applied to the participant by using an external force generator device (KineStage, Afio Corporation, Sapporo, Japan) that can control the direction, magnitude, and speed of the external force (Fig. 3a). The participant was positioned prone on the bed with the dominant arm positioned at 90° of shoulder flexion and instructed to grasp the attachment of the external force generator device. The participant’s trunk and pelvis were fixed to the bed using a belt. The direction of the external force was defined as the lower direction (the external forces were directed from the cranial to the caudal direction) (Fig. 3b). The magnitude of the external force was set at 25% of the maximum voluntary isometric contraction torque (25% MVC torque) of the shoulder flexion for each participant. We set the speed of the external force to approximately 10 N/s because the participant maintained the shoulder position against an external force during isometric shoulder contraction. In this study, the protocol consisted of rest, isometric shoulder flexion against an external force of 5 N, and isometric shoulder flexion against an external force of 25% MVC torque. First, the participant was asked to maintain the arm position at rest for 3 s. Then, the participant performed isometric shoulder flexion against an external force of 5 N for 1 s because they had to maintain the shoulder position during isometric shoulder contraction. Finally, the participant performed isometric shoulder flexion against an external force of 25% MVC torque for 5 s. Three trials were performed for isometric shoulder flexion. The participants were allowed to rest for 1 min between trials to avoid fatigue. When all three trials of the throwing arm were completed, every participant performed testing of the non-throwing arm.

**Fig. 4:**
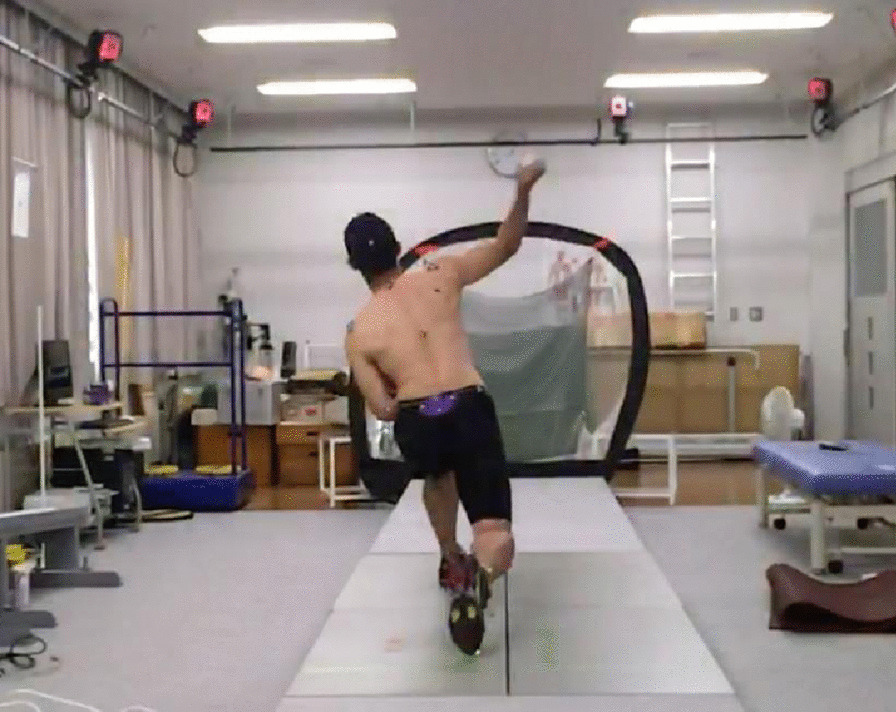
Assessment of scapular motion during baseball pitching. The participant is asked to pitch three fastballs, aiming at the center of the net located 5 m ahead of the foot of the non-throwing side

In assessing scapular motion during baseball pitching, all participants performed usual warm-ups before the pitching data measurement. These warm-ups consisted of exercises such as static and dynamic stretching, throwing exercises, simulated pitching motion, and pitching motion. After warm-ups, the participant was asked to pitch three fastballs, aiming at the center of the net located 5 m ahead of the foot of the non-throwing side (Fig. 4). The participants were allowed to rest for 1 min between the trials to avoid fatigue.

### Data analysis

Joint angles were analyzed using motion analysis software (Visual3D, C-Motion, Germantown, MD). Inverse values of scapula and humerus data were used for the left-handers to get them in line with the right-handed data. In assessing scapular control during isometric shoulder flexion, the scapular internal rotation angle, downward rotation angle, and posterior tilt angle at the start of the resting position were measured to determine the baseline. Moreover, the maximum and minimum values of the scapular internal rotation angle, downward rotation angle, and posterior tilt angle were measured from the start of the resting position to the instant at which the external force of 25% MVC torque was applied to the upper extremity. In the present study, we defined the amount of change in the scapular angle during isometric shoulder flexion as the ability to control the scapula. First, the value measured by subtracting the scapular angles at the baseline from the maximum value of the scapular angles was defined as the amount of change in the scapular internal rotation angle, downward rotation angle, and posterior tilt angle. Second, the value measured by subtracting the scapular angles at the baseline from the minimum value of the scapular angles was converted to an absolute value, and this absolute value was defined as the amount of change in the scapular external rotation angle, upward rotation angle, and anterior tilt angle. The representative values were calculated as the mean of the amount of change in the scapular internal/external rotation angle, downward/upward rotation angle, and posterior/anterior tilt angle in three trials for each participant. Finally, the side-to-side differences were calculated by subtracting the amount of change in the scapular angles of the non-throwing arm from the amount of change in the scapular angles of the throwing arm. The representative values were calculated as the mean of the side-to-side differences in three trials for each participant. In the pilot study, we tested the intra-rater reliability of the amount of change in the scapular angles of 10 healthy subjects (10 men; age, 20.9 ± 1.4 years; height, 1.73 ± 0.06 m; body mass, 67.0 ± 11.5 kg) and found that intraclass correlation coefficients (1, 3) ranged from 0.55 to 0.89. This result is detailed in Table 1.

**Table 1:** Intra-rater reliability of the amount of change in the scapular angles during isometric shoulder flexion

Scapular angle	ICC (1, 3)	95% confidence interval	
		Lower	Upper
Downward rotation	0.89	0.68	0.97
Internal rotation	0.79	0.41	0.94
Posterior tilt	0.81	0.46	0.95
Upward rotation	0.72	0.20	0.92
External rotation	0.55	0.28	0.88
Anterior tilt	0.80	0.43	0.95

*ICC*, intraclass correlation coefficient

In assessing scapular motion during baseball pitching, all kinematic data were normalized from the point of the stride foot contact to that of the ball release to a 100% scale. Foot contact was defined as the instant at which the amount of vertical ground reaction force was greater than 20 N on the foot of the non-throwing side. MER was defined as the instant at which the minimum value of the shoulder internal rotation angle was recorded. Further, we defined the middle point between two markers on the ball as the ball center and measured the distance between the wrist marker and the ball center (ball-wrist distance). To define ball release, the mean value and standard deviation of the ball-wrist distance were recorded from 300 frames before MER to the point of MER. Ball release was defined as the instant at which the ball-wrist distance was greater than the value obtained by adding the mean value and five times the standard deviation of the ball-wrist distance. Scapular and shoulder angles at MER were determined. The representative values were calculated as the mean of the scapular and shoulder angles in three trials for each participant.

### Statistical analysis

The Shapiro–Wilk test was used to determine whether the variables followed a normal distribution. Some of the data had a non-normal distribution. The paired t- and Wilcoxon signed-rank tests were used to compare the amount of change in the scapular angles (internal/external rotation angle, downward/upward rotation angle, and posterior/anterior tilt angle) during isometric shoulder flexion between the throwing and non-throwing arms. Pearson’s and Spearman’s correlation coefficients were used to determine the strength of the relationship between the amount of change in the scapular angles (internal/external rotation angle, downward/upward rotation angle, and posterior/anterior tilt angle) during isometric shoulder flexion and the scapular angles (internal rotation angle, downward rotation angle, and posterior tilt angle) at MER during baseball pitching. The strength of the relationships between correlations was defined as detailed by Portney and Watkins as follows: 0.00–0.25 = little or no relationship, 0.26–0.50 = fair degree of relationship, 0.51–0.75 = moderate to good relationship, and 0.76–1.00 = good to excellent relationship [26]. All data were analyzed using SPSS statistical software (IBM Corp., Armonk, NY). The level of statistical significance was set at *p* < 0.05.

## Results

Demographic information of the participants is presented in Table 2. The data of muscle strength are presented in Table 3.

**Table 2:** Demographic information of the participants

Variable	
Age (years)^a^	20.2 (1.9)
Height (m)^a^	1.76 (0.05)
Body mass (kg)^a^	73.3 (6.7)
BMI (kg/m^2^)^a^	23.6 (1.4)
Throwing arm (dominant hand)	4 left; 11 right
Baseball experience (years)^a^	11.5 (2.3)
Pitching experience (years)^a^	9.1 (3.1)

*BMI*, body mass index

^a^Values are presented as mean (standard deviation)

**Table 3:** Muscle strength of shoulder flexion

Variable	Throwing arm	Non-throwing arm	*p*-value
Maximum isometric muscle strength (N/kg)	3.3 (0.5)	3.0 (0.7)	.145
Maximum isometric torque (Nm/kg)	1.1 (0.2)	1.0 (0.2)	.190

Values are presented as mean (standard deviation)

All values are normalized for body mass

**Fig. 5:**
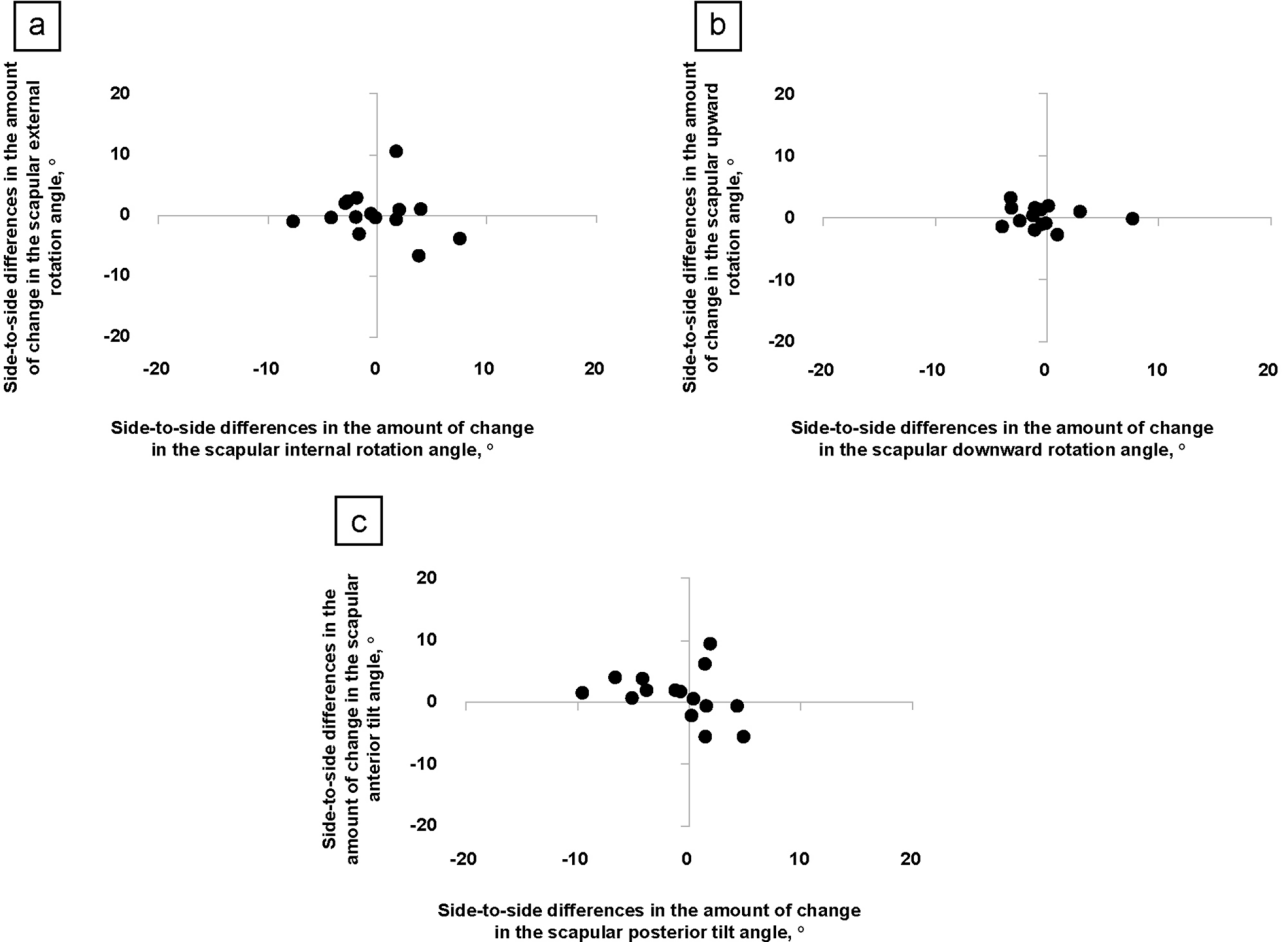
Side-to-side differences in the amount of change in the scapular angles during isometric shoulder flexion. **a** The side-to-side differences in the amount of change in the scapular internal/external rotation angle, **b** the scapular downward/upward rotation angle, and **c** the scapular posterior/anterior tilt angle

The amount of change in the scapular angles during isometric shoulder flexion is shown in Table 4. Significant differences were not detected in any of the scapular angles during isometric shoulder flexion between the throwing and non-throwing arms. Figure 5 presents the side-to-side differences in the amount of change in the scapular angles during isometric shoulder flexion.

**Table 4:** Amount of change in the scapular angles during isometric shoulder flexion

Scapular angle (°)	Throwing arm	Non-throwing arm	*p*-value	95% confidence interval	
				Lower	Upper
Internal rotation^a^	5.2 (3.4)	5.4 (3.4)	.789	− 2.3	1.0
Downward rotation^a^	6.2 (3.7)	6.8 (2.4)	.401	− 2.3	1.8
Posterior tilt^b^	3.1 (4.1)	5.7 (6.5)	.650	− 3.3	1.2
External rotation^b^	2.5 (3.3)	3.0 (2.0)	.865	− 1.8	2.4
Upward rotation^b^	1.9 (1.7)	2.0 (0.9)	.691	− 0.7	1.1
Anterior tilt^a^	4.9 (2.4)	6.0 (3.7)	.286	− 1.1	3.3

^a^Values are presented as mean (standard deviation)

^b^Values are presented as median (interquartile range)

**Fig. 6:**
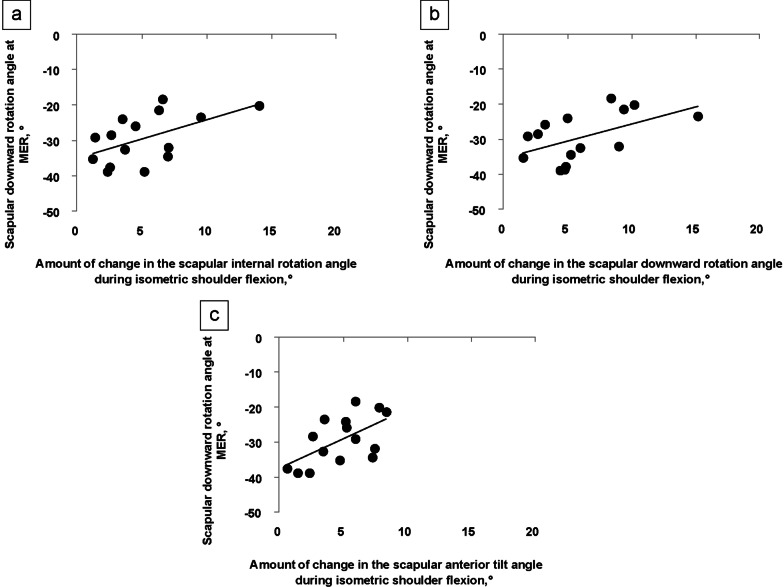
Relationship between the scapular angle during isometric shoulder flexion and scapular angle at MER. **a** The amount of change in the scapular internal rotation angle (r = 0.54, *p* < 0.05), **b** scapular downward rotation angle (r = 0.52, *p* < 0.05), and **c** scapular anterior tilt angle (r = 0.58, *p* < 0.05) during isometric shoulder flexion has a significant relationship with the scapular downward angle at MER. Abbreviation: *MER*, maximum shoulder external rotation

The scapular and shoulder angles at MER during baseball pitching are shown in Table 5. The amount of change in the scapular internal rotation angle (*r* = 0.54, *p* < 0.05), scapular downward rotation angle (*r* = 0.52, *p* < 0.05), and scapular anterior tilt angle (*r* = 0.58, *p* < 0.05) during isometric shoulder flexion had a significant relationship with the scapular downward rotation angle at MER (Fig. 6a–c). A moderately strong relationship was observed between scapular control during isometric shoulder flexion and scapular position at MER. All Pearson’s and Spearman’s correlation coefficients are listed in Table 6. The power analysis was performed using G*power software with alpha level set to 0.05 and effect size of 0.80. As a result of the power analysis, the power was 0.82 in the paired t- and Wilcoxon signed-rank tests and ranged from 0.54 to 0.66 in the Pearson’s and Spearman’s correlation coefficients.

**Table 5:** Scapular and shoulder angles at MER during baseball pitching

Variable	
*Scapular angle (°)*	
Internal rotation (+)/External rotation (−)	9.3 (10.5)
Downward rotation (+)/Upward rotation (−)	− 29.3 (6.9)
Posterior tilt (+)/Anterior tilt (−)	13.6 (8.4)
*Shoulder angle (°)*	
Horizontal adduction (+)/Horizontal abduction (−)	4.3 (9.2)
Depression (+)/Elevation (−)	− 94.8 (9.9)
Internal rotation (+)/External rotation (−)	− 130.1 (11.5)

Values are presented as mean (standard deviation)

*MER*, maximum shoulder external rotation

**Table 6:** All data of the Pearson’s and Spearman’s correlation coefficients

	Scapular angles at MER		
	Internal rotation	Downward rotation	Posterior tilt
*Scapular angles during isometric shoulder flexion*			
Internal rotation^a^	− 0.10	0.54^*^	0.09
	(.734)	(.039)	(.762)
Downward rotation^a^	− 0.16	0.52^*^	− 0.09
	(.568)	(.047)	(.737)
Posterior tilt^b^	0.28	0.06	− 0.04
	(.321)	(.820)	(.899)
External rotation^b^	0.43	− 0.03	− 0.05
	(.114)	(.930)	(.850)
Upward rotation^b^	0.19	− 0.06	0.10
	(.491)	(.830)	(.732)
Anterior tilt^a^	− 0.19	0.58^*^	0.32
	(.505)	(.023)	(.241)

*MER*, maximum shoulder external rotation

*Significant correlation (*p* < 0.05)

^a^Values are presented as the Pearson’s correlation coefficients (*p*-value)

^b^Values are presented as the Spearman’s correlation coefficients (*p*-value)

## Discussion

To our knowledge, this is the first study to quantify scapular control during isometric shoulder flexion. In healthy baseball pitchers, we showed that there was no significant difference in scapular control, as measured via isometric shoulder flexion, between the throwing and non-throwing arms. Moreover, we found a relationship between scapular control during isometric shoulder flexion and scapular position at MER during baseball pitching.

Our results showed that there was no difference in scapular control between the throwing and non-throwing arms, which is consistent with our first hypothesis. This symmetry can be attributed to the absence of side-to-side differences in isometric shoulder flexion strength [18]. At the individual level, the side-to-side differences in the amount of change in the scapular angles between the throwing and non-throwing arms ranged from -9.6° to 10.6°. However, the acromion marker cluster method has a measurement error rate up to 9.5°. Our results may not be generalizable to all collegiate baseball pitchers. Further studies are required to understand the side-to-side differences in scapular control during isometric shoulder flexion at the individual level. In this study, the amount of change in the scapular internal rotation angle, downward rotation angle, and anterior tilt angle during isometric shoulder flexion ranged from 4.9° to 6.2°. The scapula was not completely fixed to the thoracic cage during isometric shoulder flexion at 25% MVC torque in healthy baseball pitchers. In clinical settings, we suggest that evaluating the scapular position using the clinician’s manual resistance during isometric shoulder flexion should be incorporated in the screening test for pitchers.

Moreover, it has been theoretically recognized that pitchers with decreased scapular control may have abnormal scapular kinematics during pitching [8]. In our study, we found that the amount of change in the scapular downward rotation angle, internal rotation angle, and anterior tilt angle during isometric shoulder flexion had a significant relationship with the scapular downward rotation angle at MER during baseball pitching, which partially confirms our second hypothesis. This finding suggests that scapular control during isometric shoulder flexion is associated with scapular position at MER of pitching in healthy baseball pitchers. The clinicians could consider using isometric shoulder flexion to assess scapular control in baseball pitchers. During isometric shoulder flexion, the participants were required to respond to an external force and stabilize the scapula to the thoracic cage. Previous studies have shown that the trapezius and serratus anterior muscles play a crucial role in stabilizing the scapula in the directions of scapular external rotation, upward rotation, and posterior tilt during isometric shoulder flexion [27–31]. In the late cocking phase of baseball pitching, the scapula must move in the direction of upward rotation [32, 33]. The co-contraction of the trapezius and serratus anterior muscles creates a force couple of scapular upward rotation at MER during pitching [34, 35]. Pitchers with decreased scapular control who participated in this study may have weakness of the trapezius and serratus anterior muscles. It is generally believed that scapular control exercises to stimulate co-contraction of the trapezius and serratus anterior muscles improve scapular control in baseball pitchers. In the long term, further research is required to understand whether scapular control exercises affect scapular control during isometric shoulder flexion and scapular motion during baseball pitching.

This study had a few limitations. First, we did not measure periscapular muscle activity in this study. Further research is warranted to determine whether weakness of the trapezius and serratus anterior muscles affects scapular control during isometric shoulder flexion and scapular motion during baseball pitching. Second, scapular control during isometric contraction was not measured at different shoulder positions. We need further research on the relationship between scapular control during isometric contraction at different shoulder positions and scapular motion during pitching. Third, the inter-rater reliability of scapular external rotation during isometric shoulder flexion was low. The alternation in the shape of soft tissues caused by deltoid muscle contraction is a significant factor affecting the accuracy of the acromion marker cluster [24]. Moreover, normal scapular motion during shoulder movement encompasses the variability of an individual. In particular, there is wide variability in the scapular internal/external rotation during shoulder flexion [5]. Finally, we did not randomize isometric shoulder flexion trials between the throwing and non-throwing arms.

## Conclusions

No side-to-side difference was noted in scapular control during isometric shoulder flexion in healthy collegiate baseball pitchers at the group level. Further studies are required to understand the side-to-side differences in scapular control during isometric shoulder flexion at the individual level. Additionally, our results showed a relationship between scapular control during isometric shoulder flexion and scapular position at MER during baseball pitching in healthy collegiate baseball pitchers. These findings suggest that clinicians may consider using isometric shoulder flexion to assess scapular control in baseball pitchers.

## Data Availability

The datasets generated and analyzed during the current study are not publicly available due to ethical restrictions but are available from the corresponding author on reasonable request.

## Abbreviations

3D: Three-dimensional
AC: Acromioclavicular
25% MVC torque: 25% Of the maximum voluntary isometric contraction torque
MER: Maximum shoulder external rotation
ICC: Intraclass correlation coefficient
